# Special Issue: Bovine Viral Diarrhea Virus and Related Pestiviruses

**DOI:** 10.3390/v12101181

**Published:** 2020-10-19

**Authors:** Helle Bielefeldt-Ohmann

**Affiliations:** 1 Australian Infectious Diseases Research Centre, The University of Queensland, St. Lucia, QLD 4072, Australia; h.bielefeldtohmann1@uq.edu.au; 2 School of Veterinary Science, The University of Queensland, Gatton Campus, QLD 4343, Australia; 3 School of Chemistry and Molecular Biosciences, The University of Queensland, St. Lucia, QLD 4072, Australia

The genus *Pestivirus*, encompassing small positive-strand RNA viruses in the family *Flaviviridae*, comprises four viruses of very significant economic impact to the cattle, swine and sheep industries worldwide: bovine viral diarrhoea virus (BVDV) type 1 and type 2, classical swine fever virus (CSFV) and border disease virus (BDV). Both BVDV- and CSFV-related disease syndromes have been recognised for over 70 years and major progress has been made in elucidating the pathogenesis of these important infections of ruminants and pigs. While much research effort rightfully has gone into epidemiology, diagnostics and prevention—and continues to do so—BVDV and CSFV have also served as excellent models for understanding mechanisms in RNA-virus biology [1,2], transplacental virus infection [3], and host responses to persistent virus infection [4,5]. More recently, a number of novel viruses have been detected in wild and domestic animals by isolation and/or virome studies, and which appear to be related to the pestiviruses and qualify as new pestivirus species. Much still remains to be learned about these latter viruses, including host spectrum, virus–host interactions, epidemiology and clinical spectrum. 

This Special Issue of *Viruses* encompasses a range of reports on various aspects of pestiviruses, including an overview of the bovine pestiviruses with an update on the current—albeit still not widely used—nomenclature of the pestiviruses [6]. The epidemiology of pestiviruses remains a subject of considerable interest, as it impinges on biosecurity, susceptibility to secondary infections, management strategies, including vaccination versus eradication, and the importance of potential wildlife reservoirs [7,8,9,10,11]. 

A first and fundamental step in a viral infection, is attachment to a cellular membrane molecule, a viral receptor. In this issue, two articles focus on the purported receptor for BVDV, bovine CD46 [12,13], describing new tools for the study of the interaction between BVDV and CD46 and suggesting, perhaps not surprisingly considering what we know about receptors for other viruses [14], that CD46 may be but one of two or more molecules on the target cell surface membrane necessary for viral uptake [12].

The interaction of pestiviruses with the host immune system has been a focus of research for decades, notably for BVDV. In this issue the effect of congenital (transplacental) BVDV infection and persistence on the development of innate and adaptive immune functions is explored in an experimental infection model [15]. Monocytes and macrophages are known target cells in both acute-transient [16] and persistent [17,18] BVDV infections. Given the central role of these cells in both the innate and adaptive immune responses, one might expect profound and potentially adverse effects, and this is explored in one article in this issue [19].

One of the characteristics of the pestiviruses, at least the ruminant pestiviruses, is the lack of strict species specificity [6,20]. This can present challenges with regard to serology-based diagnostics, biosecurity and eradication programmes [6,20]. It may also disguise the appearance and diagnosis of new and emerging pestivirus infections, and it is therefore paramount that we gain a better understanding of these new entities and refine diagnostic capabilities—both at the clinical and the virological level. In this issue, several articles focus on three of these new pestiviruses and the diseases they may cause in pigs and ruminants: LINDA virus [21], atypical porcine pestivirus (APPV or Pestivirus K), which by now has been detected in many parts of the world [22,23,24,25], and Bungowannah virus (Pestivirus F) [26,27,28].

It is the hope that this issue, which has brought together contributions from multiple disciplines—virology, immunology, veterinary clinical medicine, epidemiology and pathology—will stimulate further exploration of this fascinating group of viruses in the future. There remains many questions to be addressed even in those very same areas dealt with in this issue, including the conditions, at both the cellular and organismic level, that are conducive to events leading to biotype-switch, viral persistence, emergence of new pestivirus diseases as well as some of the discrepancies between in vitro and in vivo results in regard to immune responses—to mention just a few. Development of new approaches to the investigation of “old” and new pestiviruses are already under way [12,13,29] and others are likely to become available in the future.
